# Effect of CDCA5 on Proliferation and Metastasis of Triple Negative Breast Cancer Cells under shRNA Interference Technology

**DOI:** 10.1155/2022/9038230

**Published:** 2022-06-11

**Authors:** Yilin Li, Wei Peng, Yunfang Deng, Jianfu Heng, Yang Yang, Xia Jin, Jigang Li, Ting Li

**Affiliations:** ^1^Department of Pathlology, Hunan Cancer Hospital, Changsha, 410013 Hunan Province, China; ^2^Department of Breast Surgery, Hunan Cancer Hospital, Changsha, 410013 Hunan Province, China; ^3^Center of New Drug Clinical Trial, Hunan Cancer Hospital, Changsha, 410013 Hunan Province, China

## Abstract

**Objective:**

It was to explore the effect of cell division cycle associated 5 (CDCA5) under shRNA interference on proliferation and metastasis of triple negative breast cancer (TNBC) cells.

**Methods:**

MDA-ME-231 and BT549 cells were selected as the research objects. According to the different interference methods and CDCA5 interference sequences, they were divided into the interference group 1_MDA-ME-231_, the interference group 2_MDA-ME-231_, the interference group 1_BT549_, the interference group 2_BT549_ (using shRNA technology), the control group _MDA-ME-231_, and the control group _BT549_ (breast cancer cells under normal culture conditions). MCF10A cells were routinely cultured as the negative control group to analyze the effect of CDCA5 expression on the proliferation and migration of cancer cells.

**Results:**

The expression of CDCA5 protein in _MDA-ME-231_ and _BT549_ cells in control group was significantly higher than that in negative control group (*P* < 0.05). Compared with the control group, the inhibition rates of CDCA5 expression in 1_MDA-ME-231_, 2_MDA-ME-231_, 1_BT549_, and 2_BT549_ cells in the interference group were 39.01%, 42.98%, 49.57%, and 60.98%, respectively (*P* < 0.05). From 12 h, the proliferation level of TNBC cells at different culture time was lower than that of the control group (*P* < 0.05). Compared with the number of staining cells in the control group, the positive staining cells in 1_MDA-ME-231_ (61.42%), 2_MDA-ME-231_ (72.06%), 1_BT549_ (52.53%), and 2_BT549_ (59.65%) in the interference group were significantly decreased (*P* < 0.05).

**Conclusion:**

The results show that the expression of CDCA5 in TNBC is increased, which plays an important role in the proliferation and migration of cancer cells. shRNA interference technology can knock down the expression of CDCA5 and inhibit its “promoting cancer” effect.

## 1. Introduction

Breast cancer, as the second most common cancer worldwide, has a very high incidence in the female population and a high mortality rate [1]. Statistics from cancer centers in China show that breast cancer is the cancer with the highest prevalence in women in China, accounting for about 17% of all cancers and a mortality rate of about 6% [2]. The incidence of triple negative breast cancer (TNBC) in all types of breast cancer is about 20% [3]. TNBC cells have deficiencies in estrogen/androgen receptor (ER/AR), and human epidermal growth factor receptor 2 (HER2) expression [4]. TNBC has rapid development and unsatisfactory chemotherapy effect, with high recurrence rate and mortality rate [5]. Therefore, it is very important to explore new effective treatments for TNBC patients as well as clinicians.

Cell division cycle associated 5 (CDCA5) is a coding gene on human chromosome 11 [6]. CDCA5 is widely expressed in tissues and organs such as bone marrow, testis, lymph nodes, bladder, lung, breast, and stomach in humans [7]. CDCA5 is very similar to gene expression of Cdk1, cyclin B, Bub 1, etc., in terms of regulation of the cell cycle [8]. Some studies showed that the role of CDCA5 in the regulation of cancer cells is mainly reflected in promoting cancer development [9]. However, The Cancer Genome Atlas (TCGA) database indicates that compared with the expression levels of RNA and protein in normal tissues, the expression levels of CDCA5 in tumor tissues are significantly different; for example, CDCA5 expression is positive in more than 70% of non-small cell lung cancer [10], CDCA5 expression is high in urothelial carcinoma, oral squamous cell carcinoma, prostate cancer, and gastric cancer, and patients have poor prognosis [11]. However, the role of CDCA5 in more cancer cells requires further exploration.

At present, the role of CDCA5 in breast cancer cells has not been revealed. However, studies suggested that the expression of CDCA5 in cancer cells of TNBC patients is significantly higher than that of normal breast cells, and patients with high expression of CDCA5 have poor prognosis [12]. Thus, studies found that TNBC cell proliferation, migration, and tumor were inhibited by RNA interference with CDCA5 [13]. RNA interference technology refers to a molecular biological technology that uses endogenous or exogenous double-stranded RNA (dsRNA) to mediate the degradation of specific mRNA in target cells and knock down or silence the expression of target genes [14]. Compared with other technologies (such as traditional antisense technology and ribozyme gene silencing method), RNA interference technology has high specificity, better transfection effect, and shorter consumption time. It has become a new means of gene function research and gene therapy research, and has been widely used in inhibiting the invasion and metastasis of tumor cells [15].

In summary, shRNA interference technology will be used to affect the expression of CDCA5, explore the effect of CDCA5 expression on the proliferation and metastasis of TNBC cancer cells, and find new potential targets for the subsequent clinical treatment of TNBC.

## 2. Materials and Methods

### 2.1. Experimental Materials

TNBC cell lines (MDA-ME-231, BT549), human normal breast cell lines (MCF10A), and pcDNA3.1 expression vectors (Strain Preservation Center); restriction enzymes, competent cells, and T4 ligase (Thermo Fisher Scientific); fetal bovine serum, trypsin, and cell culture medium (Thermo); protein transfer PVDF membrane, Western luminescence development reagent, CDCA5 antibody, and *α*-Tubulin antibody (Sigma).

### 2.2. CDCA5 shRNA Plasmid Construction

Using the information in PubMed and Sigma data, a human-derived CDCA5-specific shRNA interference sequence was designed, and two of the sequences with high scores and suitable for the pLKO1 vector were selected (Tables 1 and 2).

The single-strand primers of shCDCA5 were synthesized and diluted, and then PCR amplification was carried out. The 5 *μ*L pcDNA3.1 plasmid was treated with EcoR I and AgeI endonucleases, and the digested vector was added into 1% agarose gel. After gel electrophoresis, the digested product was recovered. PCR products, digested vector fragments, T4 ligase, buffer, and distilled water were mixed and ligated at room temperature for 2 h; 30 *μ*L of competent cells was placed on ice, and 5 *μ*L of connecting products was added to them. After ice bath, heat shock, and ice bath, the cells were cultured overnight. It was coated on the culture plate, inverted culture, and monoclonal colonies were selected, using double digestion and sequencing methods for product validation.

### 2.3. Construction of pcDNA3.1-CDCA5 Plasmid

Plasmid sequencing was performed by searching the CDCA5 gene sequence data using the NCBI website to find the CDS region information of the gene, selecting the pcDNA3.1 empty vector and the common EcoR I and BamH I restriction sites of the CDCA5 gene, and designing the primer sequences as shown in Table 3 and sending them to Invitrogen for synthesis.

The CDCA5 sequence was amplified using the PCR technical system, followed by double digestion (EcoR I and Bamh I) of the CDCA5 fragment and pcDNA3.1-puro empty vector; the CDCA5 fragment was ligated with the digested vector; the ligation product was transformed into competent cells as described above; after culture according to the method in Section 2.2, monoclonal colonies were taken, and the bacterial solution was used and sent to the company for sequencing to identify the ligation product.

### 2.4. Grouping Method

MDA-ME-231, BT549, and MCF10A cells were cultured in DMEM complete medium (10% FBS + double antibody) in a constant temperature cell incubator (37°C, 5% CO_2_ concentration). Then, breast cancer cells (MDA-ME-231 and BT549) were divided into the interference group _MDA-ME-231_ and the interference group _BT549_ according to the intervention method (shRNA technology was used to interfere with CDCA5 of breast cancer cells). According to the different CDCA5 interference sequences, the interference group _MDA-ME-231_ and the interference group _BT549_ were divided into interference group 1_MDA-ME-231_, interference group 2_MDA-ME-231_, interference group 1_BT549_, and interference group 2_BT549_. Control group _MDA-ME-231_, control group _BT549_ (breast cancer cells under normal culture conditions), and MCF10A cells were routinely cultured as negative control group. The effect of CDCA5 expression on the proliferation and migration of cancer cells was analyzed.

### 2.5. Western Blot Detection

Western blot was used to detect the CDCA5 content in the three groups of cells. The detection instruments included protein transfer PVDF membrane (GE Healthcare), Western luminescence developer (Vigorous), and electrophoresis solution and transfer solution (Dingguo, Beijing). In addition, 30% acrylamide, PH8.8/PH6.8 trihydroxyaminomethane-hydrochloric acid (Tris·HCl), sodium dodecyl sulfate (SDS), 10% ammonium persulfate (APS), tetramethylethylenediamine (TEMED) (Biomed), protein inhibitor (Cocktail, PI), and bovine serum albumin (BSA) (Roche) were used as reagents in the detection. Cell lysates were separated by SDS-PAGE, transferred to PVDF membrane, and washed with RIPA lysate. Immunostaining adopted specific primary antibodies. Then, chemiluminescence detection was carried out. The fluorescence signal was collected by Image Lab software (v4.1), and the quantification of Western blot was analyzed by Image J (v1.48).

### 2.6. Detection Test of Tumor Cell Proliferation Ability

1. The cells were digested and treated with trypsin, and counted using trypan blue staining. Each cell was seeded in a 96-well plate at a density of 2,000 cells per well, seeded in 3 duplicate wells, and seeded in a total of 7 96-well plates with 200 *μ*L of DMEM complete medium per well
2. CellTiter96 Aqueous One Solution Cell Proliferation Assay kit was placed at 4°C from -20°C in advance, thawed and dispensed into 1.5 mL EP tubes, and stored at 4°C in the dark
3. When the 96-well plate was tested, the old medium was removed, and 120 *μ*L medium (100 *μ*L serum-free medium +20 *μ*L detection solution) was added. Each plate was added with duplicate wells of 120 *μ*L medium of three uncultured cells as blank control
4. The absorbance at a wavelength of 490 nm was measured in a microplate reader after 2 h

### 2.7. Tumor Cell Migration Ability Detection Experiment

1. Trypsin-treated cells were resuspended in serum-free medium. Trypan blue staining was used to count viable cells. 500 *μ*L DMEM complete medium was added into 24-well plate. The transwell chamber was immersed in DMEM complete medium. The cells were seeded into each chamber with 2 × 104 200 *μ*L cells
2. After 24 h, the samples were collected, the samples were clearly labeled, and the cells in the chamber layer were gently erase cells in the inner chamber with cotton swabs
3. The chamber was immersed in 4% paraformaldehyde and fixed for 20 min, and the inner layer of chamber was gently dried with a cotton swab
4. The chamber was immersed in 0.2% crystal violet staining for 20 min, and the inner layer of chamber was gently dried with a cotton swab
5. The chamber was immersed in distilled water three times for 3 min, and the inner layer of chamber was gently dried with a cotton swab. A phase contrast microscope was used to analyze and read the data

### 2.8. Statistical Methods

SPSS 22.0 statistical software was applied for statistical analysis. All measurement data were expressed as mean ± standard deviation ($\overset{¯}{\text{x}}$±s). Independent sample *t*-test or rank sum test was adopted for comparison. *P* < 0.05 indicated the difference had statistical significance.

## 3. Results

### 3.1. Expression of CDCA5

#### 3.1.1. Comparison of Breast Cancer Cells and Normal Cells

**Figure 1:**
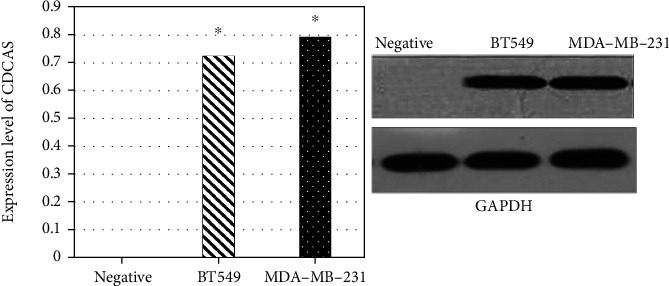
Expression of CDCA5 in breast cancer cells and normal cells (compared with the negative control group, ∗*P* < 0.05).

Western blot was used to detect the expression of CDCA5 protein in the negative control group and in the control group _MDA-ME-231_ and the control group _BT549_ cells. GAPDH was used as an internal reference protein in breast cancer cells. The expression of CDCA5 protein in _MDA-ME-231_ and _BT549_ cells in the control group was significantly higher than that in the negative control group (*P* < 0.05) (Figure 1).

#### 3.1.2. Results of CDCA5 Expression in Interference Group and Control Group

**Figure 2:**
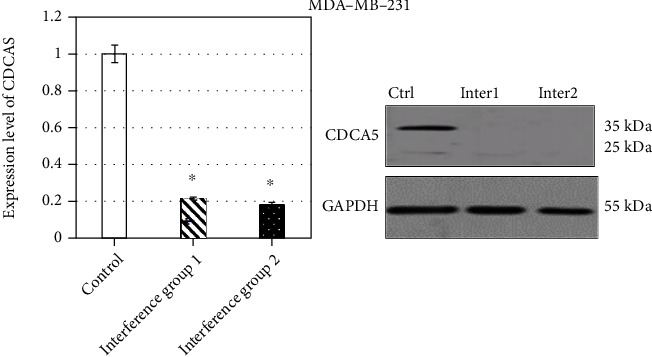
Expression level of CDCA5 in MDA-MB-231 cells (in contrast with the control group, ∗*P* < 0.05).

**Figure 3:**
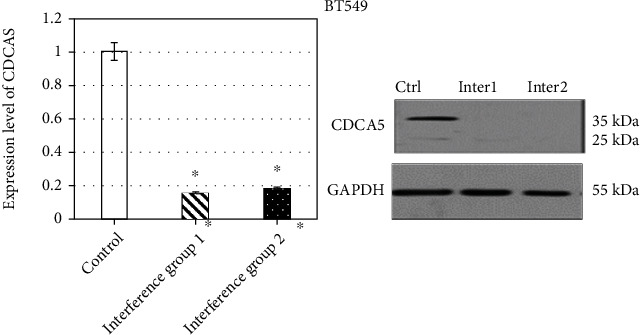
Expression level of CDCA5 in BT549 cells (in contrast with the control group, ∗*P* < 0.05).

In order to verify the effect of CDCA5 shRNA interference plasmid, the expression levels of CDCA5 in _MDA-ME-231_ and _BT549_ cells in the interference group were compared with those in _MDA-ME-231_ and _BT549_ cells in the control group. The results showed that both CDCA5 shRNA interference plasmid sequences could effectively reduce the expression level of CDCA5 in MDA-MB-231 cancer cells, and compared with that in _MDA-ME-231_ cells in the control group, the inhibition rate of 1_MDA-ME-231_ in the interference group was 39.01%, and the inhibition rate of 2_MDA-ME-231_ in the interference group was 42.98% (*P* < 0.05) (Figure 2). The effect of two CDCA5 shRNA interference plasmids on the expression of CDCA5 in BT549 cells was also consistent with the former. Compared with the control group _BT549_, the inhibition rates of CDCA5 expression in the interference group 1_BT549_ and the interference group 2_BT549_ cells were 49.57% and 60.98% (*P* < 0.05), which further provided support (Figure 3).

### 3.2. Effect of CDCA5 Expression Level on the Proliferation of Cancer Cells

**Figure 4:**
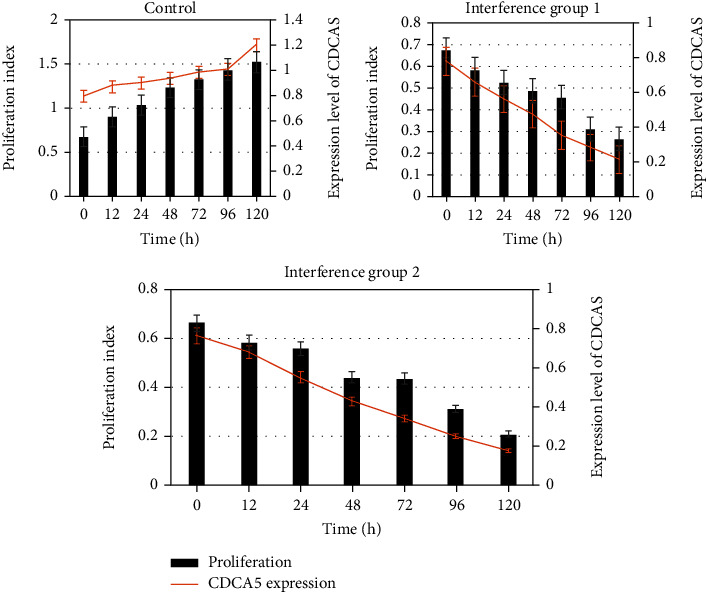
Effect of CDCA5 on proliferation of MDA-MB-231 cells.

**Figure 5:**
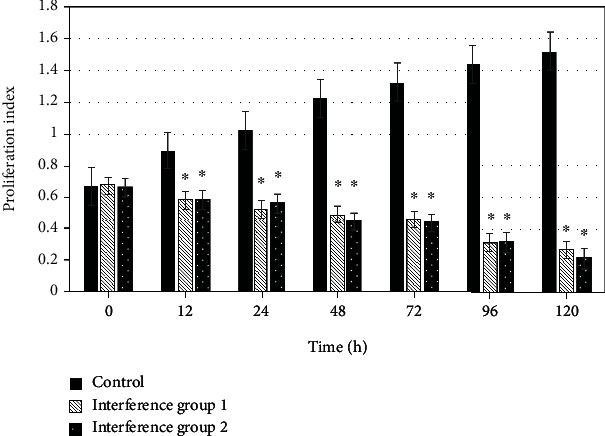
Effect of CDCA5 on the proliferation of MDA-MB-231 cells (compared with the control group, ∗*P* < 0.05).

**Figure 6:**
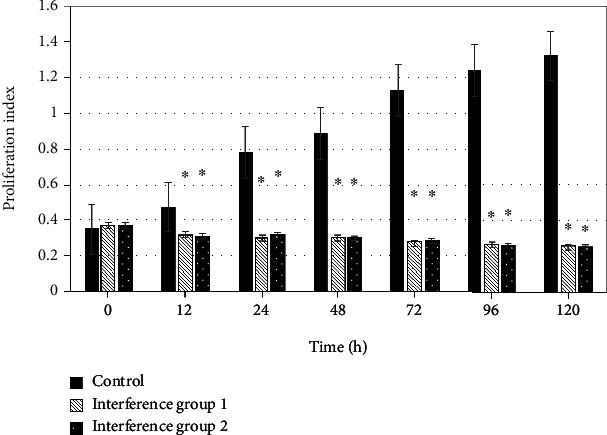
Effect of shCDCA5 on the proliferation ability of BT549 cells (compared with the control group, ∗*P* < 0.05).

Cell viability test was performed to detect the proliferation effect of MDA-MB-231 and BT549 cells at different culture time points, which was compared with the expression of CDCA5 in cells at the time points.  Figure 4 indicates the comparison of CDCA5 expression level and MDA-MB-231 cell proliferation parameters at 0 h, 12 h, 24 h, 48 h, 72 h, 96 h, and 120 h. The proliferation parameters of MDA-MB-231 cells are positively correlated with the expression level of CDCA5. The lower the expression of CDCA5, the lower the proliferation of cancer cells. Moreover, after comparison, from 12 h, the proliferation parameters of cancer cells in the interference group 1_MDA-ME-231_ and the interference group 2_MDA-ME-231_ were lower than those in the control group _MDA-ME-231_ (*P* <0.05) (Figure 5). In order to further clarify the effect of CDCA5 on the proliferation of TNBC cells under shRNA interference, TNBC cell line BT549 was rechecked, and the results were consistent with MDA-MB-231 (Figure 6).

### 3.3. Effect of CDCA5 on Metastasis of TNBC Cells

**Figure 7:**
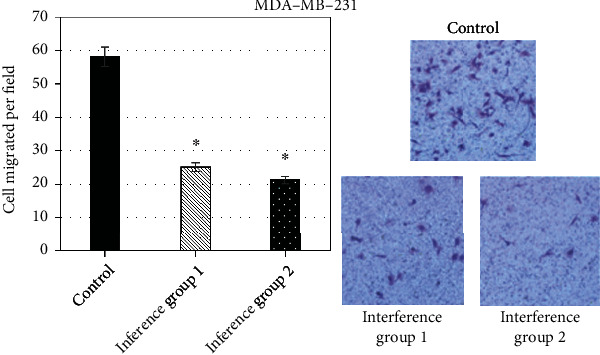
Effect of shCDCA5 on the migration ability of MDA-ME-231 cells (in contrast with the control group, ∗*P* < 0.05).

To examine the effect of CDCA5 on the migratory and invasive ability of TNBC cells, a Transwell assay was performed, which was one of the standard experiments to detect cell migration. The results presented the mean number of cells per field in the controls was 51, and the average number of 1# and 2# interference cells was 20 and 18, respectively. After knockdown of CDCA5, the number of cells per field decreased by 61.42% and 72.06%, respectively, indicating that knockdown of CDCA5 expression could significantly inhibit MDA-MB-231 cell migration (Figure 7).

**Figure 8:**
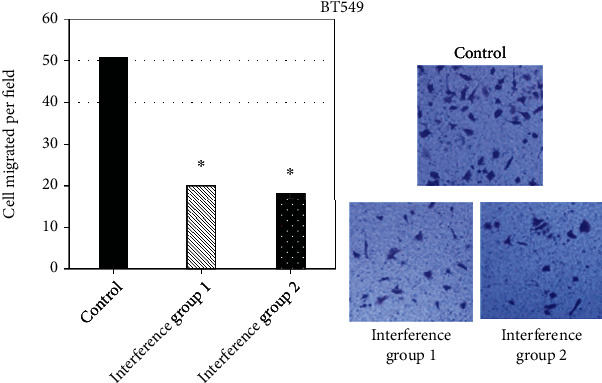
Effect of shCDCA5 on the migration ability of BT549 cells (relative to the control group, ∗*P* < 0.05).

To further confirm the phenomenon, repeated validation was carried out in TNBC cells BT549. Similarly, after interference with CDCA5 expression, the number of cells able to cross the hyaline membrane was significantly reduced, by 52.53% and 59.65%, respectively (Figure 8), suggesting that knockdown of CDCA5 was able to significantly inhibit the migration ability of TNBC cells.

## 4. Discussion

TNBC, as a type of breast cancer with high invasiveness and rapid progression, has been the focus of exploratory research in the medical community [5]. It is named for the lack of expression of commonly used breast cancer markers such as ER, PR, and HER2 in cancer tissues [16]. At present, the commonly used means of clinical treatment of breast cancer are not ideal for the treatment of patients with TNBC, and the five-year recurrence rate and mortality of patients are still maintained at a high level [17]. Therefore, it is of great significance to explore and discover new gene targets for TNBC.

The role of CDCA5 in cancer has been gradually revealed, while the role of CDCA5 as an oncogene in more tumors remains to be discovered [18]. The results showed that the protein expression of CDCA5 in _MDA-ME-231_ and _BT549_ cells in the control group was significantly higher than that in the negative control group (*P* < 0.05); that is, the expression of CDCA5 in TNBC cancer cells was higher than that in normal breast cells. This is consistent with most studies [19, 20]. shRNA interference technology was adopted to deeply explore the biological function and mechanism of CDCA5 on TNBC cells. Through experimental observation, it was found that the cell viability test results showed that relative to the cells in the controls, the cell viability of MDA-MB-231 in the interference group was significantly inhibited, with an inhibition rate of 39.01% for the interference group 1_MDA-ME-231_ sequence and 42.98% for the interference group 2_MDA-ME-231_ sequence, and the distinction had statistical meaning (*P* < 0.05). The knockdown effect of CDCA5 was detected using Western blot, and the results indicated that both interference group 1_MDA-ME-231_ and interference group 2_MDA-ME-231_ sequences could effectively reduce the expression of CDCA5 in MDA-MB-231 cells. To further clarify the effect of CDCA5 on the proliferation ability of TNBC cells under shRNA interference, the experiment was repeated in the TNBC cell line BT549. The cell proliferation ability of the interference group was found to be attenuated, with an inhibition rate of 49.57% for the interference group 1_BT549_ sequence and 60.98% for the interference group 2_BT549_ sequence, and the distinction was statistically meaningful (*P* < 0.05). It revealed that interference with CDCA5 was able to inhibit the proliferation ability of a variety of TNBC cells. In addition, the results showed that with the decrease of CDCA5 expression level, the proliferation level of TNBC cells also decreased. From 12 h, the proliferation level of TNBC cells at different culture time was lower than that of the control group (*P* < 0.05). The above results suggest that shRNA interference technology can reduce the expression of CDCA5, thereby indirectly reducing the proliferation of TNBC cells. Zhou et al. [21] also pointed out that shRNA interference technology can knock down the expression of CDCA5, affecting the proliferation, migration, and apoptosis of cancer cells.

To examine the effect of CDCA5 on the migratory and invasive ability of TNBC cells, a Transwell assay was carried out, which was one of the standard experiments to detect cell migration. The results suggested the mean number of cells per field in the controls was 51, and the average number of cells in interference group 1_MDA-ME-231_ and interference group 2_MDA-ME-231_ was 20 and 18, respectively. After using shCDCA5, the number of cells per field decreased by 61.42% and 72.06%, respectively, indicating that shCDCA5 expression could obviously inhibit MDA-MB-231 cell migration. In order to further confirm the phenomenon, the study conducted repeated validation in TNBC cells BT549. After using shCDCA5 expression, the number of cells able to cross the hyaline membrane was significantly reduced, by 52.53% and 59.65% for interference group 1_BT549_ and interference group 2_BT549_, respectively, indicating that knockdown of CDCA5 could obviously inhibit the migration ability of TNBC cells. This is consistent with the research results of Shen et al. [22] and Phan et al. [23].

In conclusion, with shRNA technical interference of CDCA5 in TNBC cell lines, the results showed that shCDCA5 had an inhibitory effect on its proliferation and migration ability. In the subsequent work, it will continue to conduct in-depth study on the regulatory function and related mechanism of CDCA5 in TNBC, expecting the study can help the clinical diagnosis and treatment of patients with TNBC.

## 5. Conclusion

shRNA interference technology was first adopted to verify the clues by immunohistochemistry, cell proliferation ability detection, cell migration ability detection, Transwell, and other experimental methods and preliminarily explore the regulatory function of CDCA5 on TNBC cells. The results show that the expression of CDCA5 in TNBC is increased, which plays an important role in the proliferation and migration of cancer cells. shRNA interference technology can knock down the expression of CDCA5 and inhibit its “tumor promotion” effect. The above research results provide a good basis for further exploring the regulatory function and corresponding mechanism of CDCA5 in TNBC and exploring the potential targets for clinical treatment of TNBC patients.

## Figures and Tables

**Table 1:** CDCA5 shRNA target sequence.

Primer name	Base sequence
CDCA5-1#	CCAAAGTACCATAGCCAGTTT
CDCA5-2#	GAGCAGTTTGATCTCCTGGTT

**Table 2:** CDCA5 shRNA primer sequence.

Primer name	Base sequence
CDCA5-1#-F	CCGGCCAAAGTACCATAGCCAGTTTCTCG AGAAACTGGCTATGGTACTTTGGTTTTTG
CDCA5-1#-R	AATTCAAAAACCAAAGTACCATAGCCAGT TTCTCGAGAAACTGGCTATGGTACTTTGG
CDCA5-2#-F	CCGGGAGCAGTTTGATCTCCTGGTTCTCG AGAACCAGGAGATCAAACTGCTCTTTTTG
CDCA5-2#-R	AATTCAAAAAGAGCAGTTTGATCTCCTGG TTCTCGAGAACCAGGAGATCAAACTGCTC

**Table 3:** pcDNA3.1-CDCA5 primer sequence.

Primer	Base sequence
CDCA5 F(*EcoR I*)	AGAGCTAGAGCTAGCGAATTCG AATGTCTGGGAGGCGAACGCG
CDCA5 R(*BamH I*)	CCCTCAGCGGCCGCGGATCCTT CAACCAGGAGATCAAACTGC

## Data Availability

The data used to support the findings of this study are included within the article.
